# The Imbalance of Cytokines and Lower Levels of Tregs in Elderly Male Primary Osteoporosis

**DOI:** 10.3389/fendo.2022.779264

**Published:** 2022-05-31

**Authors:** Wei Zhang, Wei Zhao, Wei Li, Qi Geng, Rui Zhao, Yungui Yang, Luyan Lv, Weiwen Chen

**Affiliations:** ^1^ Departments of Endocrinology, Qujing Affiliated Hospital of Kunming Medical University, Yunnan, China; ^2^ Department of Spinal Surgery, Dali Bai Autonomous Prefecture People’s Hospital, Yunnan, China; ^3^ Departments of Medical Administration, Qujing Affiliated Hospital of Kunming Medical University, Yunnan, China; ^4^ Department of Medical Laboratories, Qujing Affiliated Hospital of Kunming Medical University, Yunnan, China; ^5^ Departments of Geriatrics, The Third People’s Hospital of Qujing City, Yunnan, China; ^6^ Departments of Geriatrics, Qujing Affiliated Hospital of Kunming Medical University, Yunnan, China

**Keywords:** male primary osteoporosis, bone mineral density, cytokines, Treg cells, bone turnover markers

## Abstract

**Introduction:**

Osteoporosis (OP) is a debilitating disease that brings a heavy burden to individuals and society with reduced quality of life and lifespan. However, it’s frequently overlooked and poorly studied in elderly male patients. Worse still, few anti-osteoporosis drugs are effective at the prevention and treatment of osteoporosis in men. It has been reported that the cells of bone and the immune system share common progenitors, cytokines and growth factors, and that reciprocal interactions occur during health and disease. Nevertheless, the role of immune system in OP is not fully understood, especially in male patients. Therefore, this study aimed to investigate molecular alterations in immune cells in men with OP and to identify immunomodulatory strategies with potential therapeutic value.

**Materials and Methods:**

A population of 121 men aged between 51 and 80 years old was recruited. Bone mineral density (BMD) was measured at the lumbar spine L1-4 and femoral neck using dual-energy X-ray absorptiometry (DXA). Twenty people were healthy, 66 people had osteopenia and 35 people had OP. Bone metabolic markers, Th1, Th2, Tregs and immune molecules were evaluated at the time of enrollment.

**Results:**

Smoking was a risk factor for OP. C-terminal crosslinking of type I collagen (β-CTX) and the ratio of receptor activator of nuclear factor-κB ligand (RANKL) to osteoprotegerin (OPG) were higher in OP group, which had lower 25-hydroxyvitamin D [25(OH)D] levels. OP had the higher levels of IL-6 and TNF-α and lower levels of IFN-γ and IL-10. CD4^+^CD25^+^CD127^-/low^ Tregs were significantly lower in the OP group. The imbalance of Th1/Th2 cells may play an important role in the development of OP. 25(OH)D may play essential roles in maintaining bone health. The low level of Tregs is also one of the underlying immune mechanism that leads to male primary OP.

**Conclusion:**

The active function of osteoclasts and the decline in osteoblasts were characteristics of OP, and the imbalance in cytokines and lower levels of Tregs were observed in elderly male patients with primary OP.

## Introduction

Osteoporosis (OP) is a metabolic disease characterized by low bone mass and destruction of bone microstructures, resulting in a decrease in BMD and an increase in skeletal fragility (1). The prevalence of OP increased from 14.94% in 2008 to 27.96% in 2015 in China (2). Epidemiologists estimate that more than 1.1 million men worldwide will experience hip fractures each year by 2025 (3). Age-dependent male OP is an overlooked and a poorly studied yet increasingly important clinical problem.

Bone remodeling is a dynamic process that is dependent on the balance between osteoblasts and osteoclasts (4). Osteoblasts secrete bone matrix proteins and then stimulate their mineralization. Conversely, osteoclasts adhere to demineralized bone and dissolve bone matrix. An imbalance of bone remodeling is an important cause of OP. Recent studies have established that bone and immune cells share the same progenitors and are influenced by the same cytokines; they are functionally connected, and the immune system and bone metabolism influence each other (5).

OPG/RANK/RANKL is one of the most important molecular systems involved in regulating balance. OPG/RANK/RANKL is also an immune pathway that plays an important role in the differentiation and development of lymphocytes and lymphoid organs. Through the RANK-RANKL pathway, T cells lead to osteoclast generation and promote bone absorption (6). OPG is a negative regulator of osteoclast formation that competitively binds RANKL along with RANK. Due to the lack of transcriptional activation signals generated by the RANK-RANKL pathway, osteoclast differentiation and maturation are impeded, and bone absorption is inhibited, thus achieving bone protection. However, the synthesis of OPG gradually decreases with the gradual increase in age, the function of the RANK-RANKL pathway increases, and the incidence of OP increases. Other cytokines are also involved in bone remodeling, such as TNF-α, IL-1, IL-6 and IL-17, which promote bone resorption (7), and IFN-γ exhibits a complex bidirectional regulation mechanism in the bone immune system, which is not only related to osteoclast differentiation but is also involved in the formation of osteoblasts (8).

Recent studies have shown that Tregs inhibit osteoclasts, control bone absorption *in vivo*, maintain bone mass and reduce the occurrence of OP (9). Tregs directly inhibit RANKL to regulate osteoclast formation in a dose-dependent manner (10). Tregs can block osteoclast differentiation by inhibiting TGF-β1, GM-CSF, IFN-γ, IL-5 and IL-10 (11). Tregs can also interact with osteoblasts and have been implicated in bone formation by promoting the differentiation of osteoblasts directly and inhibiting the differentiation of osteoclasts (12). In addition to Tregs, Zhao et al. found that Th1 is also involved in the regulation of osteoclasts and bone resorption, and that abnormality of Th1 may contribute to bone destruction (13). The insufficient activation of the Th2/Treg and Th1/Th17, with subsequent osteoclast activation (14). However, the balance among Th1/Th2/Treg is less studied in the development of osteoporosis, and we will use this as an entry point to explore the pathogenesis of osteoporosis.

At present, there have been many studies on the pathogenesis of postmenopausal female OP, but insufficient attention has been given to primary OP in males. The purpose of this project was to determine the changes in the characteristics of bone metabolism in male patients with primary OP. This study will provide new ideas for clinical treatment of primary OP in males.

## Materials and Methods

For requirements for a specific article type please refer to the Article Types on any Frontiers journal page. Please also refer to Author Guidelines for further information on how to organize your manuscript in the required sections or their equivalents for your field.

### Study Participants

Males aged between 51 and 80 years who came to our outpatient department for physical examination from February 2018 to February 2019 were tested for vital signs and BMI to understand the general situation of the patients. Then, the questionnaire was completed to determine the patients’ sunburn duration, exercise duration, drinking duration and smoking duration and to determine their fracture history, past-history, family history, etc. Routine examinations, such as DXA, parathyroid hormone (PTH), calcium (Ca), phosphate (P), alkaline phosphatase (ALP), thyroid function, frontal and lateral chest radiographs, and ultrasound of the abdomen, thyroid and parathyroid gland, were performed to analyze the health status of patients. Suitable patients who volunteered to participate in our study were screened according to the inclusion and exclusion criteria, and after signing informed consent forms, six milliliters of venous blood were drawn to detect Tregs, cytokines and bone metabolic markers. This step was completed within 1 month after obtaining the medical examination report.

Subjects were included if (1) their exercise and sun intensity exposure were similar; (2) they were in good health according to medical history and current physical and laboratory examinations; and (3) they had a normal anatomical structure of the lumbar vertebra suitable for DXA assessment of BMD, with three measurable vertebrae. Subjects were excluded if they (1) had undergone any therapy affecting bone metabolism for more than two weeks in the three months directly preceding the study, including androgen-stimulating therapy, glucocorticoids or bisphosphonates; (2) had experienced bone fracture in the six months directly preceding the study; and (3) had a history of metabolic bone disease including hyperparathyroidism, hypoparathyroidism, other abnormal thyroid function, Paget’s disease and osteomalacia. The following data were obtained: 25(OH)D, β-CTX, N-terminal procollagen of type I collagen (P1NP), OPG, RANKL, Th1 cells, Th2 cells and Treg cells.

### BMD Measurement

Patients were grouped into three categories, namely, normal, osteopenia and OP, based on the lower DXA result on lumbar spine L1-4 and femoral neck according to the 2016 AACE/ACE Guidelines (15). BMD of the lumbar spine L1-4 and femoral neck were measured by DXA (GE, New York, USA). All scans were reviewed by an experienced radiologist. The AACE/ACE Guidelines for Classification of Osteopenia and OP were as follows: normal: BMD T-score ≥ -1.0, osteopenia: -2.5 < T-score< -1.0, OP: T-score ≤ -2.5.

### Sample Collection

Three milliliters of venous blood was withdrawn in ethylenediaminetetraacetic acid (EDTA) sterile tubes to isolate peripheral blood mononuclear cells (PBMCs) to detect Tregs within 1 hour after blood drawing. Three milliliters of venous blood was withdrawn in a serum tube, allowed to stand for 30 min and centrifuged at 1500 rpm for 10 min. The serum samples were separated, divided into aliquots, and then stored at -80°C for subsequent cytokines and bone-relevant serum parameter analysis. Fasting venous blood was collected from each subject between 8:00 and 9:00 AM. Detection of these parameters was completed simultaneously using the same reagent kits by the same technician.

### Measurement of Bone-Relevant Serum Parameters

Serum levels of OPG and RANKL (R&D, Germany) were determined by enzyme-linked immunosorbent assays (ELISAs), and β-CTX and P1NP (Immunodiagnostic Systems, Limited, UK) and vitamin D (DiaSorin, USA) were determined by chemiluminescence immunoassays. Ca (Beckman Coulter, China) was determined by the azoarsenic III method, P (Shanghai Kehua Bioengineering, China) was determined by the ultraviolet direct method, and ALP (Juchuang Technology, China) was determined by the International Federation of Clinical Chemistry (IFCC) recommended method. All these kits were used according to the manufacturer’s protocols.

### Measurement of T Cells and Tregs

Peripheral blood was diluted with PBS to the same volume, and PBMCs in diluted peripheral blood were isolated with lymphocyte separation fluid (lymphocyte separation fluid volume:diluted peripheral blood volume = 1:1). The finally about 0.5-1×10^6^ cells per tube were used for flow cytometry analysis. PBMCs were labeled with APC-anti-human CD4, Perp-anti-human CD8, PE-anti-human CD25 and FITC-anti-human CD127 antibodies (all flow cytometry reagents came from BD Biosciences by following the manufacturer’s protocols). The percentages of CD4^+^, CD8^+^, CD4^+^ CD25^+^ and CD4^+^ CD25^+^CD127^-/low^ cells were assessed by flow cytometry. The specificity of immunostaining was ascertained by the background fluorescence of cells incubated with CD25^+^ and CD127^+^ Ig isotype controls. FACSCaliber (BD Biosciences, Mississauga, Canada) was used to quantify the percentage of cells in all groups. CD8^+^Tregs had a low abundance (0.2- 2% of CD8^+^ T cells) in both the circulation and periphery in healthy controls. In comparison, the well-studied CD4^+^ Tregs comprise approximately 5-12% of the CD4^+^ T cell population. In our study, Tregs indicate CD4^+^ Treg cells.

### Measurement of Th1 and Th2

Measurements of Th1 (IL-2, TNF-α and IFN-γ) and Th2 (IL-4, IL-6 and IL-10) cytokines in the serum of patients, about 0.5-1×10^6^ cells, were determined by flow cytometry following the manufacturer’s protocols (Jiangxi Cellgene Biotech, China). Fluorescent signals were read and analyzed on a FACSCaliber flow cytometer with the help of BD FCAP Array v1.0.1 software (BD Biosciences).

### Statistical Analysis

Data are expressed as medians and ranges or numbers (percentages), the mean ± SD and the mean ± Q. For comparison among three groups, ANOVA for normally distributed data and Kruskal-Wallis for skewed data were performed, and *p* < 0.05 was considered significant. The data with significant differences were compared between groups, with the LSD-*t* test for normally distributed data (^*^ *p* < 0.05, ^**^ *p* < 0.01) and Kruskal-Wallis test for skewed data (^#^ *p* < 0.017 was considered significant, the test level needed to be adjusted for multiple pairwise comparisons of data, inspection level α’= inspection level α/times of comparison). Ordinal logistic regression as used for analyzing risk factors. ROC curves were generated to study the clinical indicators to predict the incidence of OP. Statistics were analyzed with SPSS 19.0 (SPSS, Inc., Chicago, IL, USA).

## Results

### Characteristics and BMD of the Studied Groups

**Figure 1:**
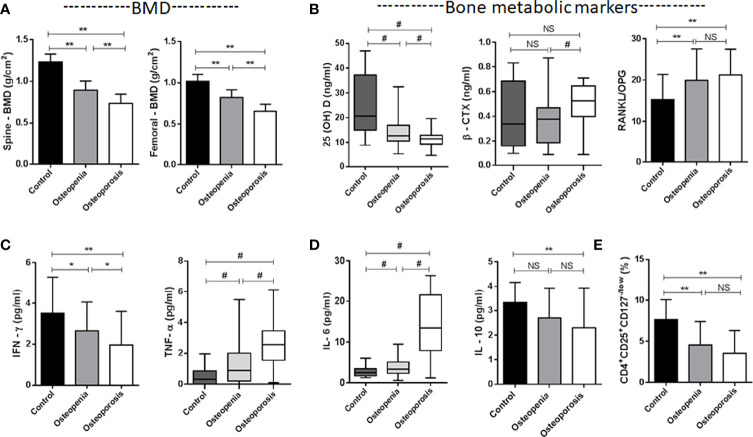
Characteristics of the studied groups. **(A)** Spine L1-4 BMD and Femoral neck BMD. **(B)** Bone metabolic markers [25(OH)VD, β-CTX, RANKL/OPG]. **(C)** Plasma levels of TNF-α and IFN-γ secreted by Th1. **(D)** Plasma levels of IL-6 and IL-10 secreted by Th2. **(E)** CD4^+^CD25^+^CD127^-/low^ Treg cells. *p < 0.05, **p < 0.01, ^#^ *p* < 0.017 (^#^ *p* < 0.017, was considered significant, the test level needed to be adjusted for multiple pairwise comparisons of data, inspection level α’= inspection level α/times of comparison). NS, No statistical differences. Data are Mean± SD or Mean ± Q.

The male patient information and clinical indicator data of the three groups are listed in **Table 1**. The smoking load in the patients with OP was significantly higher than that in the other groups. The OP group had significantly lower BMD and T scores in the lumbar spine and femur than the osteopenia and healthy control groups (**Figure 1A**). Age, milk consumption, drinking index, BMI and waist circumference in the three groups were not different. Although age had no differences in these groups, people in the OP group were over 65 years of age and were elderly. Ordinal logistic regression was used to analyze the correlation between patient information and disease development. The risk of OP was 1.005 times higher for each additional unit of the smoking index (*OR* = 1.005, 95% CI 1.003-1.007, *χ* ^2^ = 23.309, *p* < 0.001). Smoking was a risk factor for OP. To some extent, people with a high smoking index have a high incidence of OP (**Table 2**).

**Table 1:** Clinical parameters and laboratory indicators of the studied groups in males.

Parameter	Normal (*N* = 20)	Osteopenia (*N* = 66)	Osteoporosis (*N* = 35)	*Total χ* ^2^ *or F Value*	Total *p* value
**Characteristics**					
Age (Years)	62.00 ± 5.211	63.61 ± 6.194	67.40 ± 8.722	*χ* ^2^ = 5.488	0.064
≥1 serving milk (N/%)	11 (55)	29 (43.94)	16 (45.71)	*χ* ^2^ = 0.762	0.683
Drinking index	1000.00 ± 2750.00	500.00 ± 2125.00	1000.00 ± 2250.00	*χ* ^2^ = 2.582	0.275
Smoking load	304.75 ± 199.146	430.50 ± 179.799	654.86 ± 280.568	*χ* ^2^ = 27.174	0.001
BMI (kg/m^2^)	24.95 ± 1.721	25.28 ± 2.191	25.39 ± 1.899	*F* = 0.310	0.734
Waistline (cm)	87.90 ± 5.350	86.88 ± 9.181	87.89 ± 6.970	*χ* ^2^ = 0.448	0.799
L1-4 T value	0.57 ± 0.586	-1.55 ± 0.404	-3.01 ± 0.461	*F* = 396.977	<0.001
Femoral neck T value	0.45 ± 0.523	-1.64 ± 0.415	-3.06 ± 0.410	*F* = 419.067	<0.001
L1-4 BMD (g/cm^2^)	1.23 ± 0.094	0.90 ± 0.109	0.74 ± 0.110	*F* = 137.998	<0.001
Femoral neck BMD (g/cm^2^)	1.02 ± 0.083	0.82 ± 0.092	0.66 ± 0.085	*F* = 109.802	<0.001
**Bone metabolic markers**					
ALP (U/L)	88.32 ± 13.207	89.879 ± 20.828	93.03 ± 21.609	*F* = 0.428	0.653
Ca (mmol/l)	2.09 ± 0.101	2.09 ± 0.146	2.15 ± 0.129	*F* = 3.033	0.052
P (mmol/l)	1.00 ± 0.121	1.05 ± 0.261	1.10 ± 0.208	*χ* ^2^ = 2.702	0.259
25(OH)D (ng/ml)	25.22 ± 12.747	14.88 ± 6.309	11.46 ± 3.373	*χ* ^2^ = 25.143	<0.001
CTX-1 (ng/ml)	0.41 ± 0.260	0.45 ± 0.782	0.48 ± 0.192	*χ* ^2^ = 7.942	0.019
P1NP (ng/ml)	74.54 ± 29.505	73.01 ± 21.027	71.22 ± 22.113	*χ* ^2^ = 0.689	0.709
RANKL/OPG	15.29 ± 6.101	19.96 ± 7.598	21.27 ± 6.212	*F* = 4.878	0.009
**T cells**					
CD4/CD8	0.95 ± 0.400	0.97 ± 0.321	1.02 ± 0.262	*χ* ^2^ = 0.810	0.667
CD4^+^CD25^+^ (%)	14.33 ± 8.957	10.18 ± 5.077	8.94 ± 2.535	*χ* ^2^ = 5.880	0.053
CD4^+^CD25^+^CD127^-/low^ (%)	7.63 ± 2.420	4.55 ± 2.834	3.55 ± 2.754	*F* = 14.503	<0.001
**Th1 and Th2 cells**					
IL-2 (pg/ml)	0.78 ± 0.522	1.22 ± 1.628	0.97 ± 1.280	*χ* ^2^ = 0.678	0.712
TNF-α (pg/ml)	0.51 ± 0.577	1.35 ± 1.090	2.77 ± 1.433	*χ* ^2^ = 41.410	<0.001
IFN-γ (pg/ml)	3.51 ± 1.782	2.65 ± 1.400	1.96 ± 1.640	*F* = 6.537	0.002
IL-4 (pg/ml)	1.49 ± 1.070	1.26 ± 0.792	1.16 ± 0.892	*F* = 0.884	0.416
IL-6 (pg/ml)	2.75 ± 1.253	3.58 ± 1.736	13.87 ± 7.364	*χ* ^2^ = 52.433	<0.001
IL-10 (pg/ml)	3.33 ± 0.832	2.70 ± 1.230	2.30 ± 1.634	*F* = 3.947	0.022

Mean± SD or Mean ± Q or N (%)

BMI body mass index, BMD bone mineral density, ALP alkaline phosphatase, Ca calcium, P phosphate, 25(OH)D 25-hydroxyvitamin D, β-CTX C-terminal crosslinking of type I collagen, P1NP N-terminal procollagen of type I collagen, RANKL receptor activator of nuclear factor-κB ligand, OPG osteoprotegerin, IL interleukin, TNF tumor necrosis factor, IFN interferon.

1 serving milk = dairy products or cow milk were estimated to be on average 200 g or 200 ml.

Smoking load = the number of cigarettes smoked per day × the number of years smoked.

Drinking index = grams of liquor drinked per day × years of alcohol consumption (alcohol is 45-55 degrees).

**Table 2:** Analysis of risk factors for osteoporosis.

Characteristics	B	*p*	OR	95% CI for OR	
				Lower	Upper
Threshold (Normal vs other groups)	4.455	0.118	86.089	0.325	22799.930
Threshold (Normal + Osteopenia vs Osteoporosis)	7.678	0.009	2160.484	7.032	663755.146
Age (Years)	0.048	0.096	1.049	0.992	1.109
≥1 serving milk (N/%)	0.233	0.551	1.262	0.588	2.710
Drinking index	0.000	0.667	1.000	1.000	1.000
Smoking load	0.005	<0.001	1.005	1.003	1.007
BMI (kg/m^2^)	0.154	0.377	1.167	0.829	1.642
Waistline (cm)	-0.033	0.454	0.968	0.888	1.054

BMI, body mass index.

1 serving milk = dairy products or cow milk were estimated to be on average 200 g or 200 ml.

Drinking index = grams of liquor drinked per day × years of alcohol consumption (alcohol is 45-55 degrees).

Smoking load = the number of cigarettes smoked per day × the number of years smoked.

### Bone Metabolic Markers in the Studied Groups

Serum bone metabolic markers included ALP, Ca, P, 25(OH)D, β-CTX, P1NP, RANKL and OPG. ALP, Ca and P were not different among the three groups (**Table 1**). Changes in ALP in adults are common in liver diseases and severe bone tumor diseases but are not obvious in OP. In patients with OP, osteoclasts are activated to maintain blood Ca and P levels to maintain the normal electrophysiological function of cells, so blood Ca and P levels in OP had no obvious changes.

The difference in 25(OH)D was significant between groups (**Table 1** and **Figure 1B**). Although extensive studies have shown that the serum concentrations of 25(OH)D are associated with bone health and optimal overall health, a consensus has not yet been reached by a scientific community. In our study, the OP group had significantly lower expression of 25(OH)D than the other groups. Next, we will supplement vitamin D, calcium tablets and alendronate in elderly male OP patients to see if there is any improvement in OP.

β-CTX and P1NP are bone turnover markers (16). The expression of the bone resorption marker β-CTX was significantly different among the three groups (*χ* ^2^ = 7.942, *p* = 0.019) (**Table 1**). This marker was higher in the OP group, and there was a difference between the osteopenia and OP groups (**Figure 1D**). The expression of the bone formation marker P1NP was slightly lower in the OP group but did not show any differences among the groups (*χ* ^2^ = 0.689, *p* = 0.709).

RANKL and OPG are bone metabolic markers as well as immune molecules. The RANKL/OPG ratio determines the degree of bone resorption. The RANKL/OPG ratio was significantly different among the three groups (*F* = 4.878, *p* = 0.009) (**Table 1**). This parameter was higher in the OP group, but there was no difference between the osteopenia and OP groups (**Figure 1B**).

### Cytokines Secretion Profile in the Studied Groups

In our study, the impact of numerous inflammatory cytokines, including Th1 (IL-2, TNF-α and IFN-γ), Th2 (IL-4, IL-6 and IL-10), RANKL and OPG, on bone metabolism was investigated (**Table 1**, **Figures 1B–D**). The levels of IL-2 were not significantly changed in the three groups (*χ* ^2^ = 0.678, *p* = 0.712). Although IL-4 was slightly lower in the OP group, we did not observe any difference among the three groups (*F* = 0.884*, p* = 0.416). The expression levels of TNF-α (*χ^2^* = 41.410, *p<*0.001) and IL-6 (*χ* ^2^ = 52.433, *p<*0.001) in the patients with OP were significantly higher than other groups. The expression levels of IFN-γ (*F* = 6.537*, p* = 0.002) and IL-10 (*F* = 3.947*, p* = 0.022) in the OP group were lower than other groups.

This pattern in our study indicates that the high level of TNF-α (secreted by Th1) and IL-6 (secreted by Th2) in the osteoporotic patients coincides with the low level of IFN-γ and a slight low level of IL-4 (secreted by Th1) and IL-10 (secreted by Th2) in the osteoporotic patients. Th1/Th2 cell imbalance plays an important role in the development of OP. The immune molecules RANKL and OPG have been described in the previous paragraph.

### Tregs in the Studied Groups

CD4^+^CD25^+^ was previously used to detect Tregs (17, 18). With further studies, authentic Tregs highly expressed the transcription factor Foxp3 (Forkhead box protein P3), which was thought to be one of the most specific Treg cell markers (19). Foxp3 was not only a marker molecule of Tregs but also a key gene that determines the function of Tregs. However, the detection method of Foxp3 is complex. It requires cell permeabilization, preventing the isolation of viable Tregs. Subsequently, studies have found that in humans, CD127 is expressed on effector CD4+ T cells and not on Tregs. CD127 negativity or low expression can also serve as a specific marker of Tregs (20–23). There was a good correlation between high Foxp3 expression and low CD127 expression (24).

In our study, CD4^+^CD25^+^ Treg and CD4^+^CD25^+^CD127^-/low^ Tregs were simultaneously detected. CD4^+^CD25^+^ Tregs were slightly lower in the OP group, but there was no difference among the three groups (*χ* ^2^ = 5.880, *p* = 0.053). We found that CD4^+^CD25^+^CD127^-/low^ Tregs were significantly different among the three groups (*F* = 14.503, *p<*0.001) (**Table 1**). This parameter was lower in the OP group, but there was no difference between the osteopenia and OP groups (**Figure 1E**).

### Correlation Between Clinical Indicators and the Incidence of OP

**Figure 2:**
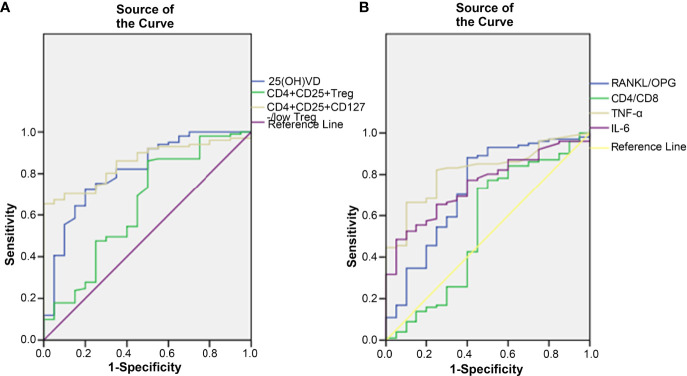
The correlation between clinical indicators and the incidence of OP. **(A)** 25(OH)D, CD4^+^CD25^+^ Treg cell and CD4^+^CD25^+^CD127^-/low^ Treg cell were found to decrease after the occurrence of OP. **(B)** RANKL/OPG, CD4/CD8, TNF-α and IL-6 were found to increase after the occurrence of OP.

ROC curves were generated to study the clinical indicators to predict the incidence of OP. In this study, 25(OH)D, CD4^+^CD25^+^ Tregs and CD4^+^CD25^+^CD127^-/low^ Tregs were found to be lower in the OP group. The AUC of 25(OH)D was 0.812 (95% CI, 0.705-0.919, *p* < 0.001), that of CD4^+^CD25^+^ Tregs was 0.649 (95% CI, 0.504-0.794, *p* = 0.036) and that of CD4^+^CD25^+^CD127^-/low^ Tregs was 0.853 (95% CI, 0.783-0.923, *p* < 0.001) (**Figure 2A**). The results showed that CD4^+^CD25^+^CD127^-/low^ Tregs and 25(OH)D were better than CD4^+^CD25^+^ Tregs at predicting the occurrence of OP. However, the positive predictive value (PPV) and negative predictive value (NPV) of 25(OH)D were better than those of CD4^+^CD25^+^CD127^-/low^ Tregs (**Table 3**).

**Table 3:** PPV and NPV of clinical indicators from the ROC curve to predict the occurrence of osteoporosis.

Test Result Variable(s)	Area	Std. Error	Asymptotic Sig.	Asymptotic 95% Confidence Interval		Cut off value	PPV	NPV
				Lower Bound	Upper Bound			
25(OH)D (ng/ml)	0.812	0.055	<0.001	0.705	0.919	14.75	94.81% (73/77)	36.36% (16/44)
CD4^+^CD25^+^ (%)	0.649	0.074	0.036	0.504	0.794	12.68	80.41% (78/97)	4.17% (1/24)
CD4^+^CD25^+^CD127^-/low^ (%)	0.853	0.036	<0.001	0.783	0.923	4.28	71.21% (47/66)	1.82% (1/55)
RANKL/OPG (%)	0.729	0.068	0.001	0.595	0.863	13.059	91.75% (89/97)	50.00% (12/24)
CD4/CD8	0.549	0.083	0.490	0.387	0.711	–	–	–
TNF-α (pg/ml)	0.817	0.044	<0.001	0.732	0.903	0.730	80.68% (71/88)	9.09% (3/33)
IL-6 (pg/ml)	0.751	0.050	<0.001	0.653	0.850	4.93	84.00% (42/50)	16.90% (12/71)

25(OH)D, 25-hydroxyvitamin D; RANKL, receptor activator of nuclear factor-κB ligand; OPG, osteoprotegerin; TNF, tumor necrosis factor; IL, interleukin.

In this study, RANKL/OPG, TNF-α, IL-6 and CD4/CD8 were found higher in OP. The AUC of RANKL/OPG was 0.729 (95% CI, 0.595-0.863, *p* = 0.001), that of TNF-α was 0.817 (95% CI, 0.732-0.903, *p* < 0.001), that of IL-6 was 0.751 (95% CI, 0.653-0.850, *p* < 0.001) and that of CD4/CD8 was 0.549 (95% CI, 0.387-0.711, *p* = 0.490) (**Figure 2B**). The results showed that TNF-α, IL-6 and RANKL/OPG were better than CD4/CD8 at predicting the occurrence of OP. The PPV and NPV of RANKL/OPG were better than those of IL-6 and TNF-α (**Table 3**).

## Discussion

Bone mass slowly decreases in men older than 60 years of age. Skeletal fragility, leading to spine and hip fractures, is a major source of morbidity and mortality. Thus, the maintenance of healthy bones is important. Bone mass is influenced by many factors, such as nutrition, physical activity, smoking and alcohol intake (25). Our study found that the smoking load in the OP group was greater than other groups, which had the lower level of plasma 25(OH)D. Smoking was a risk factor for OP. Other studies also found that serum 25(OH)D was lower in OP, and 25(OH)D may play essential roles in maintaining bone health (26, 27). In our study, there was no difference in alcohol intake among the three groups, and the proportion of elderly men who regularly drank milk was low. These results may be helpful to guide elderly men to adjust their diet and lifestyle to improve their bone health. A healthy diet including calcium, vitamin D, vitamin K and protein, regular physical activity, and not smoking help maintain bone health and delay or prevent OP (28).

DXA was used to assess BMD, and bone turnover markers were used to evaluate bone metabolism. The standard reference range for P1NP and β-CTX should not be used to diagnose OP. Our study showed that the bone turnover marker β-CTX, which represents bone resorption, was higher in the OP group, and the bone turnover marker P1NP, which represents bone formation, was slightly lower in the OP group. However, the two were not sensitive at predicting the occurrence of OP when ROC curve analysis was conducted. However, by dynamically assessing the changes in P1NP and β-CTX after treatment, the therapeutic effect of anti-OP treatments can be monitored (16, 28). Notably, elevated levels of P1NP and β-CTX predicted the presence of bone diseases associated with malignancies, including breast, prostate, and lung cancer, as well as early signs of metabolic bone disease (29–32). Therefore, P1NP and β-CTX are more useful in the differential diagnosis of OP.

Bone is a dynamic tissue that is continuously remodeled throughout life, and the remodeling process is dependent on the balance between the osteoblasts and osteoclasts. Imbalance of bone remodeling is an important cause of OP. OPG/RANK/RANKL is one of the most important molecular systems involved in regulating this balance. RANKL is considered to have the most potent resorptive effect of all osteoclastogenic cytokines (33). The result of our study revealed that RANKL/OPG was the higher in the OP group. The active function of osteoclasts and the decline in osteoblasts were characteristics of male primary OP.

Other inflammatory cytokines produced by Th1 and Th2 cells also affect bone metabolism. Some studies have indicated that TNF-α and IL-6 promote osteoclastogenesis by either inducing RANKL expression or directly acting on osteoclast precursor cells, whereas IL-10, IL-4, and IFN-γ have inhibitory effects on osteoclast differentiation and function, illustrating the tight relationship of osteoclast precursor cells with other cells in the bone microenvironment, such as innate and adaptive immune cells (34–36).

However, some studies have indicated that IFN-γ exhibits a complex bidirectional regulation mechanism in the bone immune system in which it plays both a role in bone destruction and a role in bone protection. It is not only related to osteoclast differentiation but is also involved in the formation of osteoblasts. IFN-gamma blunts osteoclast formation through direct targeting of osteoclast precursors but indirectly stimulates osteoclast formation and promotes bone resorption by stimulating antigen-dependent T cell activation and T cell secretion of the osteoclastogenic factors RANKL and TNF-alpha (8). IFN-γ has a mainly bone protective effect (37) through downregulation of osteoclast maturation (bone-degrading cells). IFN-γ can promote osteoblast differentiation and inhibit bone marrow adipocyte formation (38). Apalset et al. found an inverse association between BMD and markers of IFN-γ-mediated inflammation in the oldest participants (39). Our study suggested that the OP group had the lowest BMD and the lowest IFN-γ level. Although our study was not directly comparable with Apalset’s study, the conflicting results may indicate a difference in region or race or other reasons impacting IFN-γ activity. IFN-γ signaling as a target for the treatment of osteoporosis has been proposed (40). However, there are still many problems in the clinical application of IFN-γ, including how to control the bidirectional regulation and doses of IFN-γ, which need to be further studied.

Tregs control adaptive and innate immune responses by suppressing the activation, proliferation and function of various immune cell types, such as Th cells, Tc cells, B cells, NK cells, macrophages, and dendritic cells (41–44). Accumulated evidence has demonstrated that Tregs have the ability to suppress osteoclast differentiation *in vitro* and *in vivo*, but the mechanisms remain incompletely understood. Some studies have indicated that inhibitory cytokines or cell-cell contact dependence may be the mechanisms (45, 46). Zaiss’s group indicated that Tregs inhibit osteoclastogenesis and suppress the formation of resorption pits directly *in vitro* by CD11b^+^ monocytes that have been treated with M-CSF and RANKL (47). Our study found that Tregs was lower in OP, which was accompanied by the lower serum levels of 25(OH)D and higher serum levels of RANKL/OPG, CD4/CD8, TNF-α and IL-6 compared to the other two groups. The results of the ROC curve indicated that CD4^+^CD25^+^CD127^-/low^ Tregs and 25(OH)D were better than CD4^+^CD25^+^ Tregs at predicting the occurrence of OP. CD4^+^CD25^+^CD127^-/low^ Tregs were more sensitive and specific than CD4^+^CD25^+^ Tregs in predicting OP. However, the PPV and NPV of 25(OH)D were better than those of CD4^+^CD25^+^CD127^-/low^ Tregs.

Although some relationships between bone and the immune system have been recognized, communication factors between the different immune cell types and the bone microenvironment are still incompletely understood. We will select a matched population for a prospective study. The differentiation and maturation of osteoclasts were inhibited by alendronate. The observation group will be given supplemental calcium, vitamin D and alendronate, while the control group will only be given supplemental calcium and vitamin D. We will observe the changes in the RANK/RANKL/OPG system, inflammatory factors and CD4^+^CD25^+^CD127^-/low^ Tregs to study the effects of alendronate. The aim is to provide immunological evidence for the administration of bisphosphonates in elderly male patients with OP.

There were several limitations to this study. The bone metabolism index, Th1, Th2, Tregs and OPG/RANK/RANKL can be determined in many tissues of the body, but this study only measured the expression of the above indicators in peripheral blood, which does not fully reflect their overall role in bone metabolism. In this study, only the percentage of Tregs was measured, and the function of Tregs was not assessed.

We intend to study the bone metabolism index, Th1, Th2, OPG/RANK/RANKL and Tregs at the local bone level to verify their interrelationships in the bone microenvironment. We intend to test the function of Tregs in the future to determine the role of Tregs in OP.

## Conclusion

A healthy diet including calcium, vitamin D, vitamin K and protein, regular physical activity, and not smoking help maintain bone health and delay or prevent OP. The active function of osteoclasts and the decline in osteoblasts were characteristics of male primary OP. P1NP and β-CTX should not be used to diagnose OP, but they are useful in the differential diagnosis of OP. An imbalance in cytokines and lower levels of Tregs were observed in elderly male patients with primary OP. In this study, 25(OH)D, CD4^+^CD25^+^ Tregs and CD4^+^CD25^+^CD127^-/low^ Tregs were found to be lower in the OP group. CD4^+^CD25^+^CD127^-/low^ Tregs and 25(OH)D were better than CD4^+^CD25^+^ Tregs at predicting the occurrence of OP. The results showed that RANKL/OPG, TNF-α, IL-6 and CD4/CD8 were higher in OP. TNF-α, IL-6 and RANKL/OPG were better than CD4/CD8 at predicting the occurrence of OP. However, whether there are correlations between the RANK/RANKL/OPG system and inflammatory factors and Tregs in male patients is unclear.

## Data Availability Statement

The original contributions presented in the study are included in the article/supplementary material. Further inquiries can be directed to the corresponding authors.

## Ethics Statement

The studies involving human participants were reviewed and approved by Qujing No.1 Hospital of Yunnan Province (Qujing Affiliated Hospital of Kunming Medical University). The patients/participants provided their written informed consent to participate in this study.

## Author Contributions

YY designed the study. WZ (1st author) and performed data acquisition. WZ (2nd author) and WL conducted statistical analysis of the data. LL and YY recruited volunteers into this study. WZ (1st author), RZ and QG did research related experiments. WZ (1st author) and WZ (2nd author) wrote original draft. WC and YY reviewed and edited the article. All authors critically reviewed the paper for intellectual content and approved the final version. All authors agree to be accountable for their work and to ensure that any questions related to the accuracy and integrity of this paper will be investigated and properly resolved.

## Funding

This work was supported by Yunnan health training project of high level talents under Grant H-2019032, the Yunnan Fundamental Research Projects under Grant 2018FE001(-100) and the Scientific Research Project of Qujing Affiliated Hospital of Kunming Medical University under Grant 2019YJKT12.

## Conflict of Interest

The authors declare that the research was conducted in the absence of any commercial or financial relationships that could be construed as a potential conflict of interest.

## Publisher’s Note

All claims expressed in this article are solely those of the authors and do not necessarily represent those of their affiliated organizations, or those of the publisher, the editors and the reviewers. Any product that may be evaluated in this article, or claim that may be made by its manufacturer, is not guaranteed or endorsed by the publisher.
